# Targeting insulin-like growth factor 1 receptor restricts development and severity of secondary lymphedema in mice

**DOI:** 10.1016/j.isci.2025.111948

**Published:** 2025-02-03

**Authors:** Yinan Yuan, Sidney M. Levy, Yong Qiang Yeo, Ramin Shayan, Tara Karnezis, Steven A. Stacker, Marc G. Achen

**Affiliations:** 1 O’Brien Institute Department, St Vincent’s Institute of Medical Research, Fitzroy, VIC, Australia; 2 Peter MacCallum Cancer Centre, Melbourne, VIC, Australia; 3 Department of Nuclear Medicine, Royal Melbourne Hospital, Parkville, VIC, Australia; 4 Sir Peter MacCallum Department of Oncology, University of Melbourne, Melbourne, VIC, Australia; 5 Department of Surgery, Royal Melbourne Hospital, University of Melbourne, Melbourne, VIC, Australia; 6 Department of Medicine, St Vincent’s Hospital, University of Melbourne, Melbourne, VIC, Australia; 7 Department of Plastic Surgery, St. Vincent’s Hospital, Darlinghurst, VIC, Australia; 8 Department of Plastic Surgery, Alfred Health, Melbourne, VIC, Australia

**Keywords:** Pathophysiology, Cell biology

## Abstract

Secondary lymphedema is a debilitating chronic tissue swelling in a limb caused by inadequate interstitial fluid drainage due to dysfunctional lymphatic vessels. Pathological enlargement of small lymphatics contributes to lymphatic dysfunction in secondary lymphedema, but molecular mechanisms driving this remodeling are unclear. Here, using a surgical mouse model of secondary lymphedema and whole-genome microarray, we identified the transcript for insulin-like growth factor binding protein 5 (IGFBP5), a negative regulator of insulin-like growth factor (IGF) signaling, as the most profoundly down-regulated in lymphedematous tissue. Notably, IGF signaling via IGF1 receptor (IGF1R) was previously shown to promote lymphatic remodeling. We therefore targeted IGF1R in the mouse model using the small molecule IGF1R inhibitor linsitinib. Linsitinib restricted enlargement of small lymphatics and tissue swelling during lymphedema development, likely by inhibiting IGF1R-driven vascular endothelial growth factor-C (VEGF-C) synthesis by macrophages. Importantly, linsitinib profoundly reduced tissue swelling in mice with chronic lymphedema suggesting IGF signaling as a therapeutic target for this disease.

## Highlights

• IGF1R inhibitor linsitinib restricts development of secondary lymphedema in mice
• Linsitinib reduces tissue swelling in chronic lymphedema in mice
• Mechanism involves reduced pathological remodeling of small lymphatics
• Reduced lymphatic remodeling likely due to lower VEGF-C production by macrophages

## Graphical abstract

**Figure:**
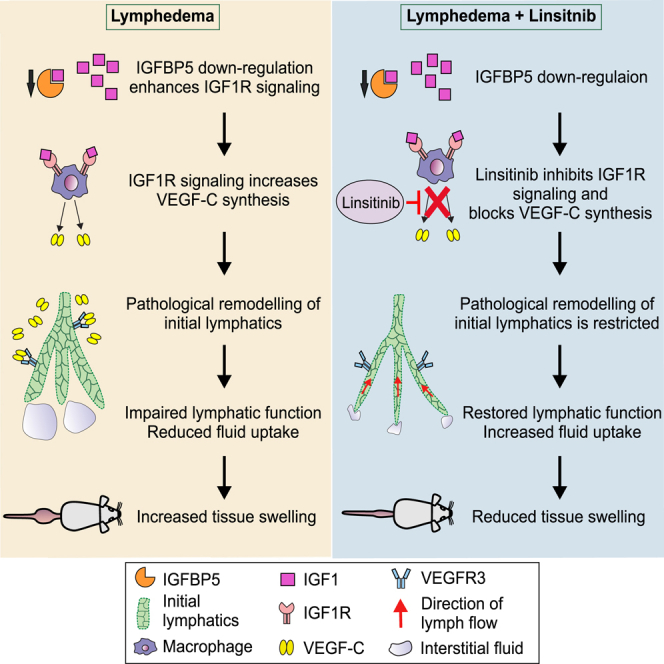

## Introduction

Secondary lymphedema is a disease characterized by debilitating, chronic tissue swelling in a limb for which a definitive cure is currently not available.^1^ It can negatively impact patient quality of life, both physically and psychologically, due to limb discomfort, anxiety, depression, sexual dysfunction, and social isolation.^2^ It is caused by impaired drainage of interstitial fluid due to functionally compromised lymphatic vessels, often as a result of surgery and/or radiotherapy for cancer.^3^ As the disease progresses, pathological lymphatic remodeling, lymph stasis, inflammation, and fibroadipose deposition can occur, all of which may exacerbate the condition.^4^^,^^5^

The dermal lymphatic system consists of distinct types of lymphatic vessels: initial lymphatics, precollector lymphatics, and collector lymphatics.^6^ Interstitial fluid is absorbed by permeable initial lymphatics in the dermis of the skin^7^ and is transported via deeper precollectors to subcutaneous collectors, which ultimately transport lymph to the venous circulation.^8^ Experimental studies of secondary lymphedema in mice demonstrated that collector lymphatics exhibit a gradual decrease of contractility during disease development, and lymphatics positive for the lymphatic marker lymphatic vessel hyaluronan receptor-1 (LYVE-1) (i.e., initial and/or precollector lymphatics^9^) become profoundly enlarged, involving active proliferation of lymphatic endothelial cells (LECs).^5^ The enlargement of initial and/or precollector lymphatics is a process of pathological lymphatic remodeling associated with gradual functional impairment likely compromising the capacity of these vessels for fluid uptake and transport.^5^^,^^10^^,^^11^ Enlargement of dermal lymphatics has also been observed in the clinical setting as assessed by lymphangiography.^12^^,^^13^^,^^14^ The molecular mechanisms driving the pathological remodeling of initial/precollector lymphatics in secondary lymphedema are unknown.

Here, we investigate molecular mechanisms, which promote the pathological remodeling of initial/precollector lymphatics, and tissue swelling, in secondary lymphedema using a surgical mouse model. Our studies indicate that the insulin-like growth factor (IGF) signaling system plays an important role in driving this lymphatic remodeling and therefore could be a relevant therapeutic target for secondary lymphedema in the clinic.

## Results

### *Igfbp5* is the most profoundly down-regulated mRNA in a mouse model of secondary lymphedema

**Figure 1:**
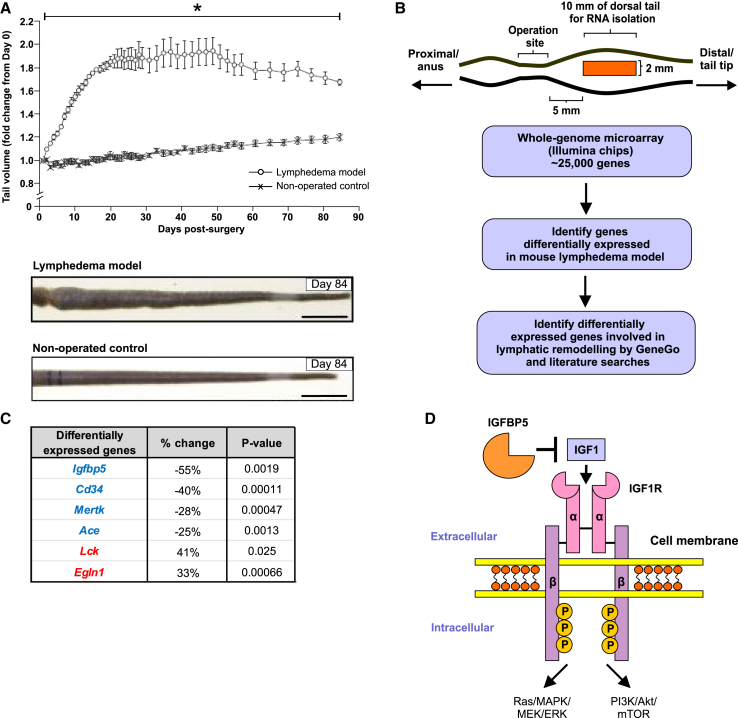
*Igfbp5* is the most profoundly down-regulated mRNA in a mouse model of secondary lymphedema Surgery employed to generate the mouse tail model is described in method details. (A) Tail swelling in the model. Top: tail volume over 3 months post-surgery. Graph shows mean ± SEM, with *n* = 6/group, and ∗*p* < 0.05 for comparison with non-operated control at each time point (Student’s t test). Bottom: representative images of tails from mouse model at 84 days post-surgery and from non-operated control; scale bars, 10 mm. (B) Analysis of the model for differentially expressed genes. Top: schematic diagram indicates location of tissue harvested for RNA analysis from tail of the model at day 84 post-surgery when lymphedema is chronic and from equivalent site of non-operated control. Bottom: flow diagram showing steps for identifying differentially expressed genes. (C) List of genes significantly differentially expressed in the mouse lymphedema model, which are involved in pathways previously shown to influence lymphatic remodeling. Expression of genes in blue was down-regulated while genes in red were up-regulated in the model compared to non-operated control. (D) Schematic diagram depicting IGF1R signaling pathway. IGFBP5 can bind IGF1 and prevent interaction of IGF1 with IGF1R. α and β denote subunits of IGF1R; “P” in yellow circles denotes phosphate group. MAPK denotes mitogen-activated protein kinase; MEK. mitogen-activated protein kinase kinase; ERK, extracellular signal-regulated kinase; PI3K, phosphoinositide 3-kinase; Akt, Ak strain transforming, and is also known as protein kinase B; mTOR, mammalian target of rapamycin.

In order to identify proteins important for pathological lymphatic remodeling in the chronic phase of secondary lymphedema, we used a mouse tail model involving surgical disruption of all lymphatics, i.e., superficial initial lymphatics, precollectors, and deep collectors (see method details for surgical details). The model resulted in a statistically significant increase in tail volume over 84 days post-surgery compared to the non-operated control (Figure 1A).

To identify differentially expressed genes, we performed whole-genome microarray analysis using RNA extracted from tail skin samples from the mouse model at day 84 post-surgery when lymphedema is chronic and compared the results to those derived from similar regions of tails from non-operated control mice (Figure 1B). These analyses showed that 149 mRNAs were altered in abundance in lymphedema, in a statistically significant fashion, compared to non-operated control (Table S1). We then used a combination of GeneGo signaling pathway analysis and literature searches to identify the subset of differentially expressed genes involved in signaling pathways, which had previously been shown to influence lymphatic remodeling. The following genes were thus identified: *Igfbp5* (encoding insulin-like growth factor binding protein 5), *Cd34* (encoding cluster of differentiation 34), *Mertk* (encoding MER proto-oncogene tyrosine kinase), and *Ace* (encoding angiotensin-converting enzyme), which were down-regulated in lymphedema, and *Lck* (encoding lymphocyte cell-specific protein-tyrosine kinase) and *Egln1* (encoding Egl-9 family hypoxia inducible factor 1), which were up-regulated (Figure 1C). *Igfbp5* was of particular interest because it was the most profoundly down-regulated gene in the lymphedema model (Table S1); the *Igfbp5* mRNA levels were reduced by 55% (Figure 1C), consistent with quantitative reverse-transcription PCR (RT-qPCR) analysis, which showed a decrease of 46% (Figure S1). Importantly, IGFBP5 can negatively regulate IGF1 signaling via IGF1 receptor (IGF1R) by binding IGF1 and thereby preventing it from activating IGF1R (Figure 1D).^15^ This negative regulation of IGF signaling by IGFBP5 is relevant to lymphatic remodeling because IGF1 signaling has been shown to promote lymphatic remodeling in mice.^16^

### Targeting IGF1R restricts development of tail swelling in a mouse model of secondary lymphedema

To study the effect of inhibiting IGF1R signaling in the lymphedema model, we treated mice daily with linsitinib (also known as OSI-906),^17^ a small molecule inhibitor of the tyrosine kinase activity of IGF1R, beginning the day of surgery until 17 days post-surgery. This time period was chosen because the rapid increase in tail volume exhibited by the model was largely complete by day 17 (Figure 1A). The dosage of 40 mg/kg/day, delivered by oral gavage, was employed based on a previous study reporting good tolerability of this dose in mice, and favorable efficacy as assessed by monitoring phosphorylation of IGF1R in the mouse liver; i.e., this dose prevented autophosphorylation of IGF1R,^18^ which is a hallmark of its activation.

**Figure 2:**
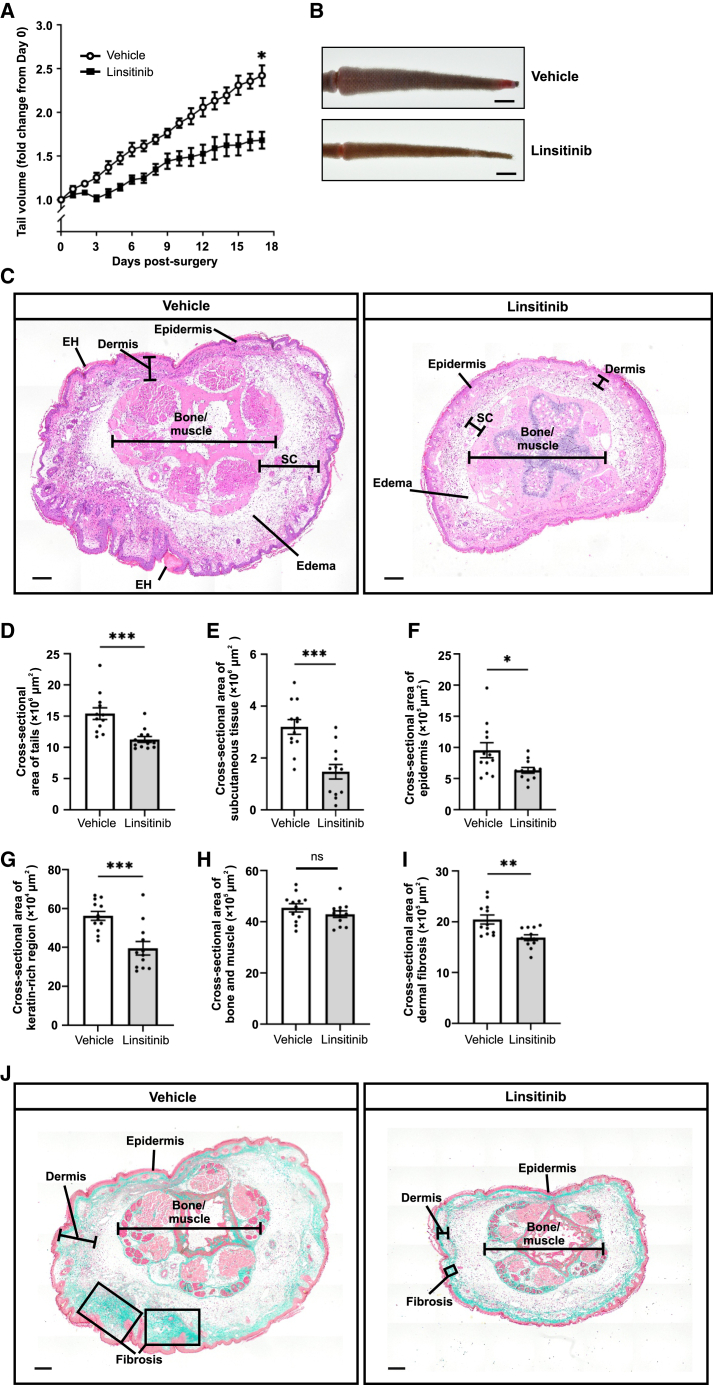
Linsitinib restricts development of tail swelling in a mouse model of secondary lymphedema Mice were subjected to surgery and daily treatment with linsitinib for 17 days beginning the day of surgery. (A) Tail volume in response to linsitinib treatment. Graph shows mean ± SEM, *n* = 12/group, ∗*p* < 0.01 for comparison with vehicle at day 17 post-surgery (Student’s t test). (B) Representative images of tails at day 17 post-surgery in the linsitinib and vehicle control groups. Scale bars, 10 mm. (C) Representative H&E-stained cross-sectional images of mouse tails from linsitinib and vehicle control groups. EH denotes epidermal hyperkeratosis, and SC denotes subcutaneous layer. Scale bars, 200 μm. (D–J) Quantification of cross-sectional area of tails (D), subcutaneous tissue (E), epidermis (F), keratin-rich region (G), and bone and muscle (H). Quantitation of dermal fibrosis by Masson’s trichrome method (see method details) (I). Representative examples of staining for Masson’s trichrome method (J). Dermal fibrosis is indicated by green staining of collagen fibers in the dermis, whereas muscle and epithelium stain red/pink. Regions of pronounced dermal fibrosis are indicated by rectangles. Scale bars in (C) and (J), 200 μm. Graphs in (D)–(I) show mean ± SEM, with *n* = 12/group; ns, not statistically significant; ∗*p* < 0.05, ∗∗*p* < 0.01, ∗∗∗*p* < 0.001 for comparison at day 17 post-surgery (Student’s t test).

Treatment with linsitinib reduced tail volume and tissue swelling compared to vehicle control throughout the 17-day treatment period, and the 31% reduction in tail volume at the pre-determined assessment time point of 17 days post-surgery was statistically significant (Figures 2A and 2B).

Histological examination of tails from the linsitinib-treated group at day 17 indicated reductions in the cross-sectional area of the tails, subcutaneous tissue, and epidermis compared to vehicle control (Figures 2C–2F). Epidermal hyperkeratosis was apparent in the vehicle control but not in the linsitinib group as indicated by monitoring keratin-rich regions (Figures 2C and 2G). There was no significant difference in the area of bone and muscle between the study groups (Figure 2H). Given that dermal fibrosis is a hallmark of clinical secondary lymphedema,^19^ we assessed if linsitinib restricted tissue fibrosis in the mouse model. Fibrosis was assessed by detecting dermal collagen fibers using Masson’s trichrome staining, which demonstrated that the linsitinib group had a reduction in the cross-sectional area of dermal fibrosis compared to vehicle control, which was statistically significant (Figures 2I and 2J). However, the reductions in the cross-sectional areas of epidermis, dermal fibrosis, keratin-rich regions, and fat, caused by linsitinib treatment, contributed less to the total decrease in the tail cross-sectional area compared to the reduction in subcutaneous tissue area, which accounted for 46% of the total decrease (Table S2). These findings demonstrate that linsitinib treatment restricted the development of multiple important aspects of lymphedema pathophysiology. In particular, the decrease in the cross-sectional area of subcutaneous tissue is consistent with a reduction in subcutaneous edema.

### Linsitinib inhibits pathological enlargement of lymphatic vessels

**Figure 3:**
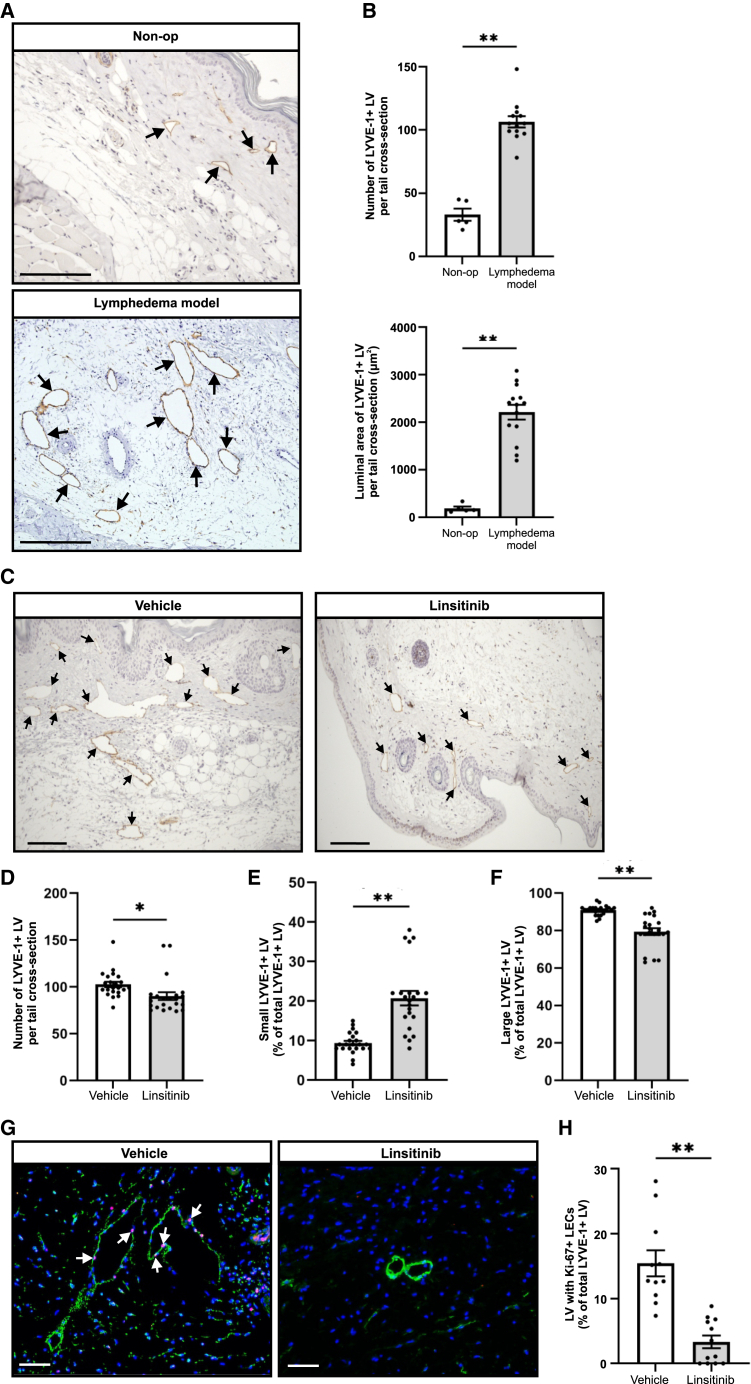
Linsitinib inhibits pathological enlargement of lymphatic vessels (A) Representative images of immunohistochemical staining for LYVE-1+ lymphatic vessels (indicated by arrows) in tail cross-sections from non-operated (Non-op) control and mouse lymphedema model at day 84 post-surgery. Scale bars, 100 μm (B) Quantification of LYVE-1+ lymphatics (top) and luminal area of LYVE-1+ lymphatics (bottom). (C) Representative images of immunohistochemical staining for LYVE-1+ lymphatic vessels (indicated by arrows) in tail cross-sections from linsitinib and vehicle control groups at day 17 post-surgery. Scale bars, 200 μm. (D) Quantification of LYVE-1+ lymphatics per tail cross-section. (E and F) Percentage of (E) small LYVE-1+ lymphatics and (F) large LYVE-1+ lymphatics relative to all LYVE-1+ lymphatics. Lymphatics were detected in the dermis and subcutaneous tissue, and no lymphatics were scored in the epidermis, bone, or muscle. The small and large lymphatics are defined using the average luminal size of LYVE-1+ lymphatics in non-operated control (170 μm^2^) (i.e., “small” are below this size and “large” are above it). (G) Representative images of immunofluorescence staining of tail cross-sections for lymphatic vessels (LYVE-1; green) and Ki-67 (pink) from linsitinib and vehicle control groups. Nuclei were stained with DAPI (blue). Examples of Ki-67+ LECs in lymphatics are indicated by arrows. Scale bars, 100 μm. (H) Percentage of LYVE-1+ lymphatics with Ki-67+ LECs. All graphs show mean ± SEM. In (B), *n* = 13 for lymphedema model and *n* = 5 for Non-op; in (D)–(F), *n* = 22/group; in (H), *n* = 12 for linsitinib and *n* = 11 for vehicle. LV denotes lymphatic vessels. ∗*p* < 0.05, ∗∗*p* < 0.001 (Student’s t test). Grubb’s outlier test was performed on data shown in (F), which demonstrated that none of the data points were outliers.

Our finding that linsitinib reduced the area of subcutaneous tissue in the lymphedema model, consistent with reduced edema, led us to focus on the effects of this drug on lymphatic remodeling. As a preliminary step, we analyzed lymphatic vessels in the mouse model, and non-operated control, 84 days post-surgery by immunohistochemistry for LYVE-1, a marker of initial and precollector lymphatic vessels.^9^ This demonstrated a statistically significant increase in the number and size of LYVE-1+ lymphatics in the dermis and subcutaneous tissue in the mouse lymphedema model (Figures 3A and 3B) (nota bene: all LYVE-1+ lymphatics in tails were detected in the dermis and subcutaneous tissue). It is thus apparent that lymphangiogenesis and lymphatic vessel remodeling occur in the mouse model.

We then monitored the effect of linsitinib on lymphatic vessels in the model (17 days post-surgery) by LYVE-1 immunostaining. Linsitinib decreased the number of LYVE-1+ lymphatics by approximately 15%, which was statistically significant (Figures 3C and 3D). Linsitinib also altered the size of these vessels causing a statistically significant increase in the proportion of small LYVE-1+ lymphatics (Figure 3E), and a decrease in the proportion of large LYVE-1+ lymphatics, compared to vehicle control (Figure 3F). Further, we demonstrated that the linsitinib group exhibited a statistically significant reduction in the proportion of LYVE-1+ lymphatics with LECs expressing the cell proliferation marker Ki-67, compared to vehicle control (Figures 3G and H). These findings demonstrate that linsitinib treatment restricted pathological lymphangiogenesis and remodeling of initial and/or precollector lymphatics, likely by inhibiting the proliferation of LECs in these vessels.

### Linsitinib decreases abundance of macrophages and VEGF-C levels in lymphedema

**Figure 4:**
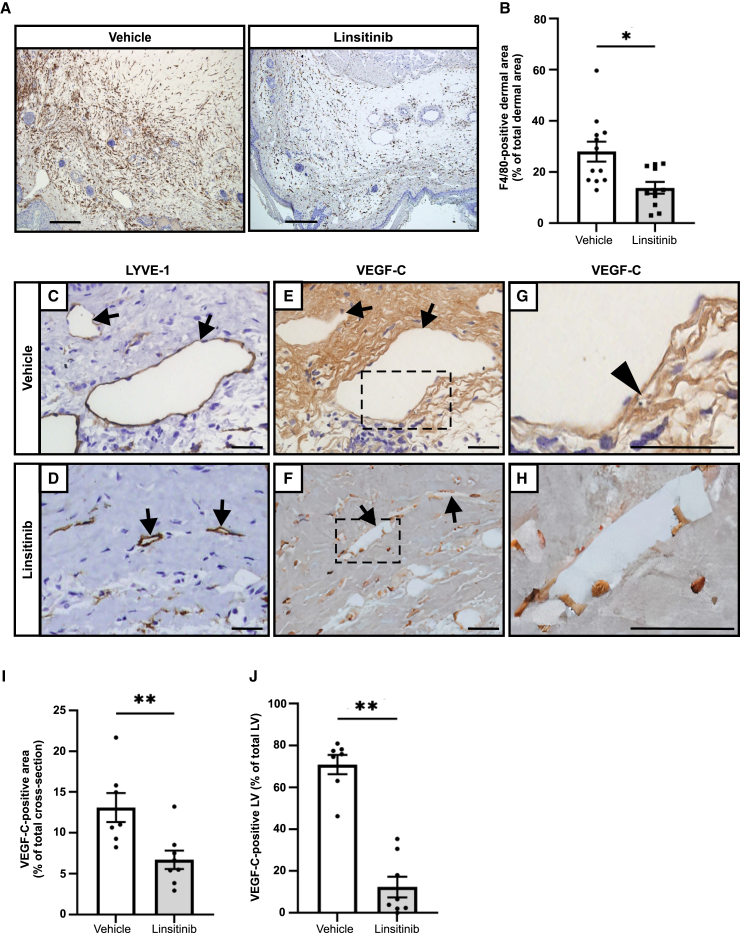
Linsitinib decreases F4/80+ macrophages and VEGF-C in a mouse model of secondary lymphedema (A) Representative images of immunohistochemical staining for F4/80-positive macrophages (brown) in tail cross-sections from linsitinib and vehicle control groups. Scale bars, 200 μm. (B) Percentage of F4/80+ dermal area relative to total dermal area in tail cross-sections. Graph shows mean ± SEM, *n* = 11 for linsitinib and *n* = 12 for vehicle control groups, ∗*p* < 0.01 for comparison at day 17 post-surgery (Student’s t test). (C–F) Representative images of immunohistochemical staining for (C and D) LYVE-1 and (E and F) VEGF-C in tail cross-sections from linsitinib and vehicle control groups. Lymphatic vessels are indicated by arrows. Sections in (E) and (F) are serial to those in (C) and (D), respectively. Scale bars, 100 μm. (G and H) Magnified images of dotted rectangles from (E) and (F), respectively. Lymphatic endothelium positive for VEGF-C is indicated by arrowhead. Scale bars, 100 μm. (I) Percentage of VEGF-C+ area relative to total tail cross-sectional area. (J) Percentage of VEGF-C+ LYVE-1+ lymphatics relative to total LYVE-1+ lymphatics (this analysis was conducted using serial sections immunostained for VEGF-C and LYVE-1 as described in method details). Graphs in (I) and (J) show mean ± SEM, *n* = 8 for linsitinib and *n* = 7 for vehicle; LV, lymphatic vessels; ∗∗*p* < 0.0001 for comparison at day 17 post-surgery (Student’s t test).

Vascular endothelial growth factor (VEGF)-C is a lymphangiogenic growth factor, which plays a critical role in development of the lymphatic vasculature.^20^^,^^21^ Previous studies demonstrated that lymphedematous tissue exhibits increased levels of macrophages expressing VEGF-C in mouse secondary lymphedema models, which is associated with increased pathological lymphatic remodeling.^22^^,^^23^ Notably, analysis of biopsies from patients with secondary lymphedema demonstrated over 3-fold increase in the number of macrophages in lymphedematous tissue compared with normal limb controls from the same patients.^22^ Hence, we sought to assess the effect of linsitinib on macrophages and VEGF-C in our mouse model of lymphedema. Immunohistochemistry was employed for this analysis as this method allows monitoring of not only the site of VEGF-C synthesis but also the distribution of VEGF-C in tissues after secretion from cells.^24^ Immunostaining for the mouse macrophage marker F4/80 demonstrated that there was a 50% reduction in the area stained by this marker in the dermal region of the linsitinib group compared to vehicle control (Figures 4A and 4B). We observed VEGF-C immunostaining on the lymphatic endothelium of large LYVE-1+ lymphatics, and in tissue adjacent to these vessels, in the vehicle control (Figures 4C, 4E, and 4G), whereas the linsitinib group exhibited weaker or no VEGF-C staining on LYVE-1+ lymphatics or in surrounding tissue (Figures 4D, 4F, and 4H). Quantification indicated that treatment with linsitinib not only reduced the VEGF-C-positive area in tails (Figure 4I) but also decreased the proportion of lymphatic vessels positive for VEGF-C compared to vehicle control (Figure 4J); these two effects were both statistically significant.

Enlargement of lymphatic vessels has been shown to be induced by VEGF-A,^25^^,^^26^^,^^27^ as well as VEGF-C; hence, we investigated whether linsitinib altered the distribution of VEGF-A in the mouse lymphedema model. Our immunohistochemical results showed that there was no detectable difference in the VEGF-A-positive areas in tails between the linsitinib and vehicle control groups (Figure S2). Immunofluorescence for the prospero-related homeobox domain1 (PROX-1), a master regulator of lymphatic vessel development and sprouting,^28^^,^^29^^,^^30^ demonstrated the presence of this protein in nuclei of LECs lining LYVE-1+ lymphatic vessels in both linsitinib and vehicle control groups (Figure S3).

**Figure 5:**
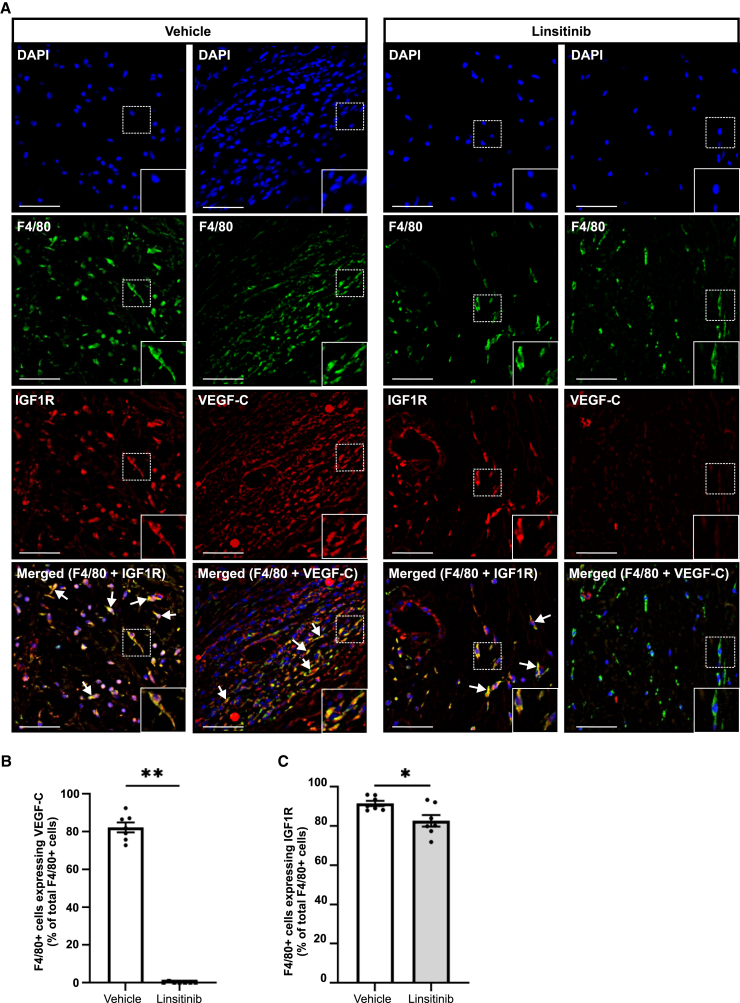
Effect of linsitinib on IGF1R and VEGF-C expression in macrophages in a mouse model of secondary lymphedema (A) Representative 60x images of immunofluorescence staining for macrophages (F4/80; green), IGF1R (red), and VEGF-C (red) in tail cross-sections from linsitinib and vehicle control groups. DAPI was used to stain cell nuclei (blue). F4/80+ macrophages, which were also positive for IGF1R or VEGF-C, are indicated by arrows. Magnified images (dotted rectangles) are inserted in the bottom right of panels. Scale bars, 50 μm (B and C) Percentage of F4/80+ cells (B) positive for VEGF-C or (C) positive for IGF1R. Graphs show mean ± SEM, *n* = 7/group. ∗*p* < 0.05, ∗∗*p* < 0.0001 for comparison at day 17 post-surgery (Student’s t test).

Importantly, immunofluorescence for both F4/80 and VEGF-C demonstrated that approximately 80% of F4/80+ macrophages were positive for VEGF-C in the vehicle control group whereas virtually none were positive for VEGF-C in the linsitinib group (Figures 5A and 5B). Linsitinib also reduced the proportion of F4/80+ macrophages that were positive for IGF1R, although only slightly, from approximately 90% to 80% (Figures 5A and 5C). IGF1R was not detected on LECs of lymphatic vessels in either the linsitinib or vehicle control groups (data not shown). However, we did detect IGF1R on cells that were not F4/80+ macrophages or LECs, and linsitinib did not detectably decrease the expression of IGF1R on these cells (Figure 5A). Overall, these findings demonstrate that linsitinib reduced the abundance of F4/80+ macrophages in the lymphedema model and almost totally blocked VEGF-C expression by these macrophages.

### Linsitinib reduces tissue swelling in established lymphedema

We demonstrated earlier that linsitinib restricts tissue swelling of the lymphedema model during the first 17 days post-surgery, when lymphedema develops in the vehicle control, which is relevant to the clinical scenario of restricting development of secondary lymphedema post-surgery for cancer. However, an alternative and important clinical need is to treat pre-existing secondary lymphedema. We therefore treated our mouse model daily with linsitinib beginning on day 30 post-surgery, a time point after tissue swelling has occurred, until day 60. Mice were then monitored until day 90 so that the response to cessation of treatment could be assessed. The pre-determined time points for statistical comparison were days 30, 60, and 90 post-surgery.

**Figure 6:**
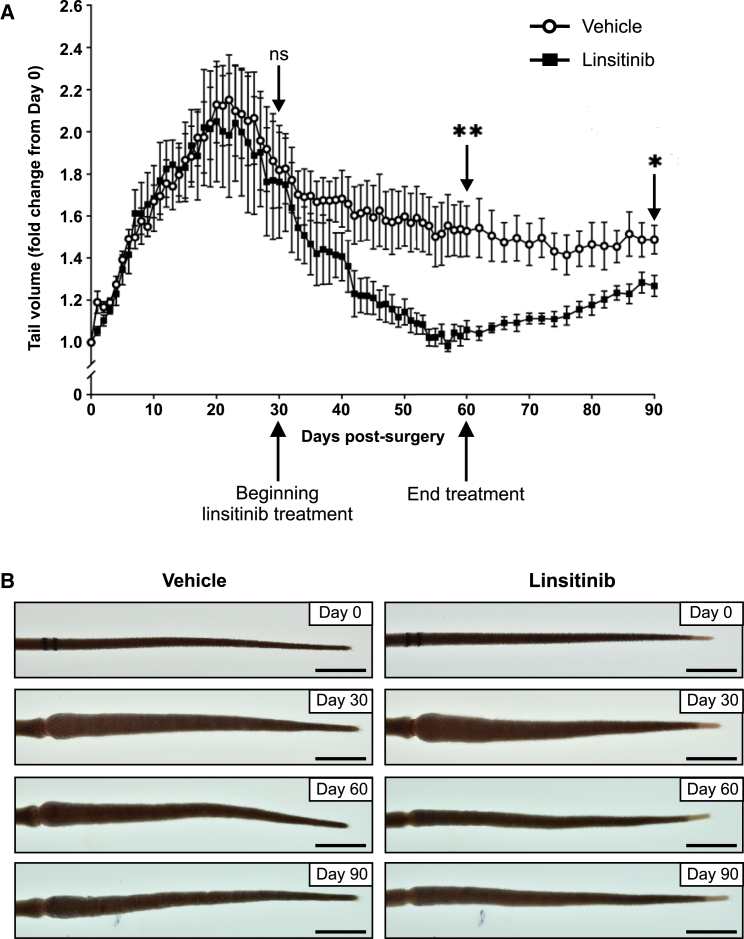
Linsitinib reduces tissue swelling in established lymphedema in the mouse model Mice were subjected to tail surgery and developed lymphedema over 30 days and were then treated with linsitinib (or vehicle control) daily from day 30 to 60 inclusive. Mice were monitored post-treatment until day 90. (A) Tail volume in linsitinib and vehicle control groups. Graph shows mean ± SEM, *n* = 5 for linsitinib and *n* = 6 for vehicle. ns, not statistically significant; ∗*p* < 0.05 and ∗∗*p* < 0.001 for comparison between linsitinib and vehicle control at the pre-defined time points of day 30 (beginning of treatment), 60 (end of treatment), and 90 post-surgery (Student’s t test). (B) Representative images of tails at days 0, 30, 60, and 90 post-surgery in the linsitinib and vehicle control groups. Scale bars, 10 mm.

Treatment with linsitinib from day 30 to 60 post-surgery was effective in reducing tail volume in the mouse model causing a 31% reduction in volume compared to vehicle control at day 60, which was statistically significant (Figures 6A and 6B). The tail volume in the linsitinib group at day 60 was reduced to a level comparable to that observed at the time of surgery. After treatment ended on day 60, tail volume in the linsitinib group gradually increased until day 90, although the volume at day 90 was still less than that for the vehicle control. These data indicate that linsitinib can reduce tissue swelling in the context of pre-existing secondary lymphedema; however, continued treatment may be necessary to maintain the benefit of reduced tissue volume.

## Discussion

Pathological remodeling of dermal lymphatics is an important aspect of clinical secondary lymphedema.^12^^,^^13^^,^^14^ Here, using a surgical mouse model of secondary lymphedema, we demonstrated that targeting IGF1R signaling with linsitinib restricted both tissue swelling and pathological remodeling of LYVE-1+ lymphatics (i.e., initial and/or precollector lymphatics) during lymphedema development. We had a specific focus on small lymphatics, as pathological remodeling of small lymphatics has been shown to contribute to lymphatic dysfunction in lymphedema.^5^ We therefore hypothesize that it would be important to enhance the function of the abnormally enlarged small lymphatics, as well as damaged collecting lymphatics, to treat lymphedema. We demonstrated here that linsitinib restricted lymphangiogenesis and enlargement of small lymphatics in our mouse lymphedema model. Our findings suggest that these effects of inhibiting IGF1R with linsitinib could be due to reduced VEGF-C levels, which is supported by previous studies demonstrating that VEGF-C can promote both lymphangiogenesis^31^ and enlargement of lymphatic vessels,^5^ and that IGF1R signaling can induce VEGF-C expression via downstream PI3K/Akt and mitogen-activated protein kinase/ERK1/2 signaling pathways.^32^^,^^33^ Although VEGF-A has been reported to promote lymphatic vessel enlargement in mice,^25^^,^^26^^,^^27^ our data indicated no detectable difference in VEGF-A distribution between the linsitinib and vehicle control groups, suggesting that linsitinib does not influence lymphatics by modulating VEGF-A expression. Our findings suggest that linsitinib restricted pathological remodeling of initial and/or precollector lymphatics by blocking IGF1R-driven VEGF-C synthesis in the mouse lymphedema model.

**Figure 7:**
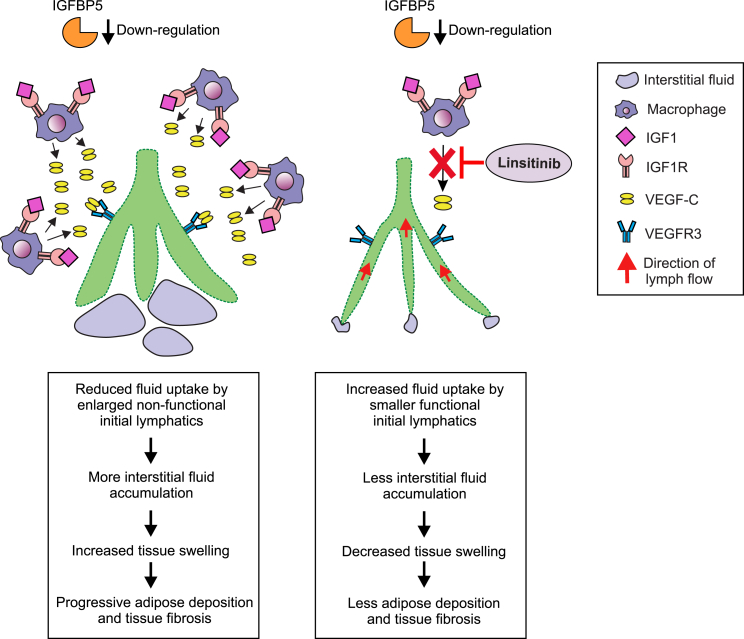
Mechanisms by which linsitinib reduces tissue swelling in secondary lymphedema Down-regulation of IGFBP5 in lymphedematous tissue promotes activation of IGF1R signaling on macrophages, which enhances production of VEGF-C leading to proliferation of LECs, pathological dilation of initial and/or precollector lymphatics, and impaired capacity for lymphatics to take up and transport interstitial fluid, resulting in excess interstitial fluid and tissue swelling (left). Treatment with linsitinib reduces the abundance of macrophages and blocks activation of IGF1R signaling in macrophages, thereby suppressing production of VEGF-C and restricting pathological lymphatic remodeling and tissue swelling (right).

Previous studies using mouse models of secondary lymphedema, and samples of human secondary lymphedema, showed increased levels of macrophages in lymphedematous tissue.^22^^,^^23^^,^^34^ In both mice and humans, these macrophages were reported to express VEGF-C.^22^^,^^23^^,^^34^^,^^35^ We showed that treatment of our mouse model with linsitinib profoundly reduced both the abundance of macrophages and VEGF-C expression in the macrophages. Notably, we found that IGF1R is expressed in most macrophages in the model, which is consistent with previous studies demonstrating the presence of mRNA for IGF1R in bone marrow-derived macrophages of mice.^36^^,^^37^ Given the localization of IGF1R on macrophages, expression of VEGF-C by these cells, and previous findings that IGF1R signaling can drive VEGF-C expression in a cell autonomous fashion,^32^^,^^33^ it is likely that IGF1R activation directly drives expression of VEGF-C by these cells. Therefore, it is probable that linsitinib directly inhibits macrophage expression of VEGF-C, although we cannot formally exclude the involvement of indirect mechanisms. The molecular mechanisms by which IGF1R promoted recruitment of macrophages in our lymphedema model are unclear; however, a likely scenario is that the initial lymph stasis after surgery in our model promotes recruitment of macrophages in an IGF1R-dependent fashion. This leads to a significant accumulation of macrophages producing VEGF-C, leading to pathological remodeling of lymphatics, which further compromises lymphatic drainage and exacerbates the condition (Figure 7).

We found that linsitinib was effective in restricting multiple histopathological aspects of lymphedema during disease development in the mouse model. More specifically, linsitinib treatment was associated with reductions in the cross-sectional areas of epidermis, subcutaneous tissue, epidermal hyperkeratosis, and dermal fibrosis. Previous studies have shown that IGF1R is expressed in epidermal keratinocytes, where stimulation with IGF1 increased proliferation of these cells.^38^^,^^39^^,^^40^ IGF1R is also expressed in fibroblasts, and these cells can secrete type I collagen in response to stimulation with IGF1,^41^^,^^42^ potentially contributing to fibrosis. Thus, linsitinib may exhibit direct effects on keratinocytes and fibroblasts to inhibit hyperkeratosis and fibrosis, respectively, in our mouse model. However, our findings indicate that the effects of linsitinib on these pathological features had less impact on tissue swelling in the model than did its effect on subcutaneous edema. Furthermore, another study reported that IGF1R is expressed in mouse LECs *in vitro* and can directly induce lymphangiogenesis independent of VEGFR-3 signaling.^16^ Although we did not detect IGF1R by immunofluorescence on the LECs of lymphatic vessels in our model, we cannot discount the possibility of low levels of IGF1R on these cells, below the level of detection of our methodology, which could contribute to lymphangiogenic signaling. Our findings that linsitinib improved multiple pathological aspects of the mouse model could be clinically significant because thickening of the epidermis and subcutaneous tissue, hyperkeratosis, and dermal fibrosis are all pathological features of secondary lymphedema in humans.^35^^,^^43^^,^^44^^,^^45^

We demonstrated here that linsitinib is useful for treating established lymphedema in our mouse model, as well as for restricting development of the condition. Treatment of established lymphedema led to a significant reduction in tail volume, which is encouraging from a clinical perspective; however, tail volume began to increase after cessation of linsitinib treatment. It is noteworthy that linsitinib demonstrated good tolerability in humans during a clinical trial for cancer in which it was administered for up to two years without any adverse events deemed due to the drug.^46^ Further, long-term IGF1R blockade in adult mice was well tolerated and had beneficial effects on aging-relevant metabolism.^47^ The favorable safety profile of targeting IGF1R raises the possibility of using linsitinib or other IGF1R inhibitors, including IGF1R monoclonal antibodies,^48^ to treat secondary lymphedema in the clinic, potentially for long periods of time. Long-term treatment of patients with IGF1R inhibitors may be required given our finding that tissue swelling recurred in our model after completion of linsitinib treatment. Overall, our findings indicate that further preclinical, and potentially clinical, development of IGF1R inhibitors for the treatment of secondary lymphedema is warranted.

### Limitations of the study

One limitation of this study is the lack of analysis of human tissue. Ideally this would involve a direct comparison between biopsies from lymphedematous human tissue and unaffected control tissue from the same patients. While such an analysis would enhance the relevance of our findings to clinical settings, it has not been feasible for us to access unaffected control tissue as surgeons are generally unwilling to remove such tissue from lymphedema patients. Another limitation is that the surgery that gives rise to clinical secondary lymphedema is typically associated with lymph node excision (i.e., lymphadenectomy).^49^ The surgical mouse tail model employed here does not involve lymphadenectomy; however, it does involve ablation of collecting lymphatic vessels similar to the effect of lymphadenectomy on the lymphatic vasculature. Finally, our microarray analysis of mRNA levels in the mouse lymphedema model was conducted at day 84 post-surgery, when chronic lymphedema had become well established, so that we could characterize molecular changes in the chronic disease state. However, it would be beneficial to examine earlier time points to further enhance understanding of the changes that drive chronic lymphedema.

## Resource availability

### Lead contact

Further information and request for resources and reagents should be directed to and will be fulfilled by the lead contact, Marc G. Achen (machen@svi.edu.au).

### Materials availability

• This study did not generate new unique reagents.
• Materials generated in this study will be shared by the lead contact upon request.

### Data and code availability

• All data reported in this paper will be shared by the lead contact upon request.
• The microarray experiment data described in this publication are available in GEO via accession number GEO: GSE256205.
• Any additional information required to reanalyze the data reported in this paper is available from the lead contact upon request.

## Acknowledgments

We thank Stephen Fox, Elena Takano, David Byrne, Sophie Paquet-Fifield, and Sally Roufail from the Peter MacCallum Cancer Centre (Peter Mac) and Jason Palmer from the O’Brien Institute Department at St Vincent’s Institute of Medical Research for technical assistance and advice. We also acknowledge Mark Devlin from the Monash Institute of Pharmaceutical Sciences and Peter Mac for assistance with analysis of linsitinib. This work was supported by grants from the 10.13039/501100000925National Health and Medical Research Council of Australia (1183926, 1053535, and 1045523 to M.G.A. and S.A.S.), a Research Fellowship from the Hummingbirds Fundraising Volunteers (to Y.Y.), an Australian Postgraduate Award from the Australian Federal Government (to Y.Y.), and funding from the O'Brien Foundation (to M.G.A, T.K., and R.S.) and the Stafford Fox Medical Research Foundation (to M.G.A., T.K., and R.S.).

## Author contributions

Conceptualization: Y.Y., S.M.L., M.G.A., and S.A.S. Methodology: Y.Y. and S.M.L. Investigation: Y.Y., S.M.L., and Y.Q.Y. Funding acquisition: M.G.A. and S.A.S. Supervision: M.G.A. and S.A.S. Writing – original draft: Y.Y. Writing – review and editing: Y.Y., S.M.L., Y.Q.Y., M.G.A., S.A.S., T.K., and R.S. All the authors read and approved the manuscript.

## Declaration of interests

M.G.A. and S.A.S. own stock in Opthea Pty. Ltd., a company developing inhibitors of VEGF proteins.

## STAR★Methods

### Key resources table

**Table:** 

REAGENT or RESOURCE	SOURCE	IDENTIFIER
**Antibodies**		

Rabbit polyclonal anti-Lyve1	Abcam	Cat# ab14917; RRID: AB_301509
Rat monoclonal anti-F4/80	Abcam	Cat# ab6640; RRID: AB_1140040
Rabbit polyclonal anti-VEGF-C	Novus Biologicals	Cat# NB110-61022; RRID: AB_925699
Rat monoclonal anti-Lyve1	ThermoFisher Scientific	14-0443-82; RRID: AB_1633414
Rabbit polyclonal anti-IGF1R	Santa Cruz Biotechnology	Cat# sc-712; RRID: AB_671788
Rabbit monoclonal anti-Ki67	Abcam	Cat# ab16667; RRID: AB_302459
Rabbit monoclonal anti-PROX1	Abcam	Cat# ab199359; RRID: AB_2868427
Goat polyclonal anti-VEGF-A	R&D Systems	Cat# AF-493; RRID: AB_354506
Goat IgG isotype control	Invitrogen	Cat# 02–6202; RRID: AB_2532946
Biotinylated goat anti-Rabbit	Vector Laboratories	Cat# BA-1000; RRID: AB_2313606
Biotinylated rabbit anti-Rat	Vector Laboratories	Cat# BA-4000; RRID: AB_2336206
Goat anti-Rat IgG (H + L) Alexa Fluor 488	ThermoFisher Scientific	Cat# A-11006; RRID: AB_2534074
Goat anti-Rabbit IgG (H + L) Alexa Fluor 568	ThermoFisher Scientific	Cat# A-11011; RRID: AB_143157

**Biological samples**		

FFPE mouse tail samples	This manuscript	N/A

**Chemicals, peptides, and recombinant proteins**		

Linsitnib (OSI-906)	Ark Pharm	N/A
Tartaric acid	Sigma-Aldrich	Cat# 251380
Target Retrieval Solution, Citrate pH 6.1 (10×)	Agilent Technologies	Cat# S1699
Proteinase K, Ready-to-Use	Agilent Technologies	Cat# S3020
Protein Block, Serum-Free	Agilent Technologies	Cat# X0909
Streptavidin/HRP	Agilent Technologies	Cat# P0397
DAPI fluoromount G	ProSciTech	Cat# IM035

**Critical commercial assays**		

Diaminobenzamine substrate kit	Vector Laboratories	Cat# SK-4100
High-Capacity cDNA Reverse Transcription Kit	Applied Biosystems	Cat# 4368814
TaqMan Fast Universal Master Mix	Applied Biosystems	Cat# 4444556
TaqMan® Gene Expression Assay for Igfbp5	Applied Biosystems	Mm00516037_m1
Mouse β-actin mRNA	Applied Biosystems	Mm00607939_s1
RNeasy Mini kit	Qiagen	Cat# 74104

**Deposited data**		

Raw microarray data	GEO	GEO: GSE256205

**Experimental models: Organisms/strains**		

Mouse: C57BL/6	Animal Resources Center	RRID:MGI:2159769

**Software and algorithms**		

ImageJ	https://imagej.net/ij/	RRID:SCR_003070
GraphPad Prism	https://www.graphpad.com/	RRID:SCR_000306
CorelDRAW Graphics Suite	https://www.coreldraw.com/	RRID:SCR_014235
HALO	https://indicalab.com/halo/	RRID:SCR_018350
MetaMorph Microscopy Automation and Image Analysis Software	https://www.moleculardevices.com/	RRID:SCR_002368

### Experimental model and study participants details

#### Mice

Six-week old female C57BL/6 mice were obtained from Animal Resources Center (Perth, Australia) to generate a surgical mouse tail model of secondary lymphedema. All animal studies reported here were approved by the Animal Experimentation Ethics Committee of the Peter MacCallum Cancer Center (approval number E562).

### Method details

#### Generation of mouse secondary lymphedema model

The mouse tail model was generated using 6-week-old female C57BL/6 mice by creating two circumferential incisions at 15 and 17 mm from the base of the tail and excising a 2 mm ring of dermis and subcutaneous tissue from the underlying connective tissue thus removing the dermal initial and precollector lymphatics. In addition, deep lymphatic collectors adjacent to the dorsolateral tail veins were identified using Patent Blue dye (Sigma-Aldrich, MO, US), and disrupted by bipolar diathermy. After surgery, the mouse was housed in a static micro-isolator on a heat pad for 24 h. After one day post-surgery, the mouse was kept at room temperature and tail volume was monitored for up to 84 days, until sacrifice for tissue analysis.

#### Tail volume measurement

Digital images of tails were analyzed with a custom-made journal, which measured serial diameters every 1.2 mm from the distal edge of the wound to the tail tip, using MetaMorph Microscopy Automation and Image Analysis Software (Molecular Devices, CA, USA). Serial diameters were inserted into the truncated cone formula (Sitzia, 1995) to calculate tail volumes.

#### RNA isolation

Tail skin samples were harvested on day 84 post-surgery, 5 mm distal from the center of the scar that formed in response to surgery, with equivalent sites excised from non-operated control mice. A 10 mm long, 2 mm wide region of skin was removed from each mouse and placed in 1 mL RNAlater stabilization solution (Qiagen, Australia) overnight, then stored at −80°C. Total RNA was isolated using a Qiagen RNeasy Mini kit, and RNA integrity was assessed using an Agilent RNAnano 6000 chip.

#### Microarray analysis

Total RNA (1 μg) was used for whole-genome microarray with the mousRef-8 v2.0 Expression BeadChip (Illumina, CA, USA) which targets 25,000 annotated transcripts. Hybridizations were performed with four biological replicates. Synthesis of biotin-labelled complementary RNA, hybridization, image acquisition and raw data generation were carried out at the Australian Genome Research Facility (Melbourne, Australia). Microarray data were log_2_ transformed and quantile normalized using Partek Genomic Suite (Partek Inc, MO, USA). Genes with a *p*-value ≤0.05 and fold change of ≥1.2 were considered statistically significant and differentially expressed (Hawkins et al., 2007; Wang et al., 2006), and were subjected to pathway analysis.

#### Gene pathway analysis

GeneGo (Thomson Reuters, CA, USA) was employed to identify signaling pathways involving statistically significantly and differentially expressed genes, and PubMed literature searches were conducted to explore the association of these genes/pathways with lymphatic remodeling.

#### Quantitative reverse-transcription PCR

Reverse transcription was performed with 3 μg of total RNA using a High-Capacity cDNA Reverse Transcription Kit (Applied Biosystems, CA, USA), and resulting cDNA was used for quantitative PCR with a StepOne Plus Instrument (Applied Biosystems), TaqMan Fast Universal Master Mix (Applied Biosystems) and TaqMan Gene Expression Assay (Applied Biosystems) for Igfbp5 (Mm00516037_m1), in triplicate. Mouse *β-actin* mRNA (Applied Biosystem, Mm00607939_s1) was the endogenous control. Relative changes in mRNA levels were assessed using the 2ˆΔΔCt method (Livak and Schmittgen, 2001).

#### Linsitinib treatment

Mice received daily treatment with 40 mg/kg of linsitinib (Ark Pharm, IL, USA) in 25 mM tartaric acid or a vehicle control via oral gavage. The purity of the linsitinib used in this study was demonstrated by liquid chromatography–mass spectrometry to be greater than 98%. The capacity of this linsitinib to inhibit autophosphorylation of IGF1R in response to IGF1 was confirmed using 3T3-L1 cells and Western blotting for phospho-IGF1Rβ (data not shown).

#### Histology

Cylindrical segments of tails, located between 2.5 and 5.0 mm distal to the center of the scar that formed in response to surgery, were excised, fixed in 4% paraformaldehyde overnight at 4°C on a rocker, decalcified and dehydrated as described (Biswas et al., 2023), except that decalcification was for 14 days. Tissues were then embedded in paraffin, cut into serial sections of 4 μm thickness and mounted onto Superfrost Plus glass slides (ThermoFisher Scientific, MA, USA). Quantification of tissue components (subcutaneous tissue, epidermis, bone, muscle and epidermal hyperkeratosis) was performed in a blinded fashion using H&E-stained tail cross-sections. Tail components were manually traced, and areas quantitated using ImageJ software. In addition, dermal fibrosis was assessed by staining collagen fibers using the Masson’s trichrome method (Schipke et al., 2017), and positively stained areas were manually traced and quantified using HALO Image Analysis Software (Indica Labs, NM, USA).

#### Immunohistochemistry

Tissue sections were dewaxed and rehydrated, and antigen retrieval was conducted using either pH 6.0 target retrieval solution (Agilent Technologies, CA, USA) for 25 min at 96°C (for LYVE-1 and F4/80 staining) or proteinase K (Agilent Technologies) for 8 min at room temperature (RT) (for VEGF-A and -C staining). After quenching peroxidase with 3% H_2_O_2_, non-specific binding was blocked with protein block (Agilent Technologies) for 30 min at RT. Sections were incubated with rabbit-*anti*-LYVE-1 (Abcam, #ab14917, 1:250, Australia), rat anti-F4/80 (Abcam, #ab6640; 1:400), goat anti-VEGF-A (R&D Systems, #AF-493, 1:200, MN, USA) or rabbit anti-VEGF-C (Novus Biologicals, #NB110-61022; 1:100, CO, USA) at 4°C overnight, then with biotinylated goat anti-rabbit IgG (Vector laboratories, BA-1000; 1:200, CA, USA) or biotinylated rabbit anti-rat IgG (Vector laboratories, BA-4000; 1:200) for 30 min at RT followed by streptavidin-HRP conjugate (Agilent Technologies) and visualization using a diaminobenzamine substrate kit (Vector Laboratories). Concentration-matched IgG isotype control (Invitrogen, 02–6202, MA, USA) was used as a negative control. Images were captured with a BX61 light microscope (Olympus, Tokyo, Japan).

#### Immunofluorescence

Immunofluorescence was performed similarly to immunohistochemistry, but without quenching peroxidase activity. Primary antibodies were rat anti-LYVE-1 (ThermoFisher Scientific, #14-0443-82; 1:100), rat anti-F4/80 (Abcam, #ab6640; 1:200), rabbit anti-IGF1R (Santa Cruz Biotechnology, #SC-712; 1:100, TX, USA), rabbit anti-Ki-67 (Abcam, #ab16667, 1:50), rabbit anti-VEGF-C (Novus Biologicals, #NB110-61022; 1:100), and rabbit anti-PROX1 (Abcam, #199359, 1:200). Following incubation with primary antibodies at 4°C overnight, sections were incubated with Alexa Fluor 488 goat anti-rat (ThermoFisher Scientific, #A-11011) or Alexa Fluor 568 goat anti-rabbit antibodies (ThermoFisher Scientific, #A-11006), and coverslipped with DAPI fluoromount G (ProSciTech, QLD, Australia). Images were captured with a BX61 Olympus fluorescence microscope.

### Quantification and statistical analysis

Initial and precollector lymphatics in tails were detected by LYVE-1 immunostaining and quantitated by manual counting conducted in a blinded fashion. Luminal sizes of LYVE-1+ lymphatics were measured using Image J software. Quantification of LYVE-1+ lymphatics expressing VEGF-C was conducted using two serial sections from each tail, each stained with LYVE-1 or VEGF-C, and only lymphatics that were present in both sections were included in the analysis.

Two-tailed Student’s t test was employed to assess statistical significance using GraphPad Prism (GraphPad Software, MA, USA). All data are presented as mean ± SEM with significance set at p < 0.05. n and p values for each experiment are provided in the figure legends.
