# Supplementary material for: Targeting insulin-like growth factor 1 receptor restricts development and severity of secondary lymphedema in mice
Source: iScience. 2025 Feb 3;28(3):111948. doi: 10.1016/j.isci.2025.111948 (PMC11889606; doi:10.1016/j.isci.2025.111948)
Supplement: Document S1. Figures S1–S3 and Tables S1 and S2 [file mmc1.pdf]

iScience, Volume *28*

Supplemental information

Targeting insulin-like growth factor 1 receptor restricts development and severity of secondary lymphedema in mice

Yinan Yuan, Sidney M. Levy, Yong Qiang Yeo, Ramin Shayan, Tara Karnezis, Steven A. Stacker, and Marc G. Achen

Table S1. List of differentially expressed genes in the mouse lymphedema model

Gene*	Fold change **	P-value	Gene	Fold change	P-value
Igfbp5	-2.18767	0.00191586	*B4galnt4*	-1.33794	0.012911
Gfpt2	-2.15984	0.000472385	*Ace*	-1.33761	0.00132181
Gsta3	-2.11992	0.000631581	*Gstp2*	-1.334	0.00388237
Tmem100	-2.10162	0.000373242	*Satb1*	-1.33172	0.0464041
Rgs7	-1.84593	0.000640807	*Wdr45l*	-1.3302	0.0128143
Pcolce2	-1.78953	0.000549353	*Hsd11b2*	-1.32795	0.00657104
Laptm4a	-1.77255	0.00283138	*Speer4b*	-1.3253	0.0100899
Pi16	-1.76456	0.0467036	*2610020C11Rik*	-1.32056	0.00069517
Clec3b	-1.73689	0.00249364	*Slc23a3*	-1.31913	0.0266555
Scara5	-1.68784	0.00480962	*Sulf1*	-1.31909	0.00868377
Galntl5	-1.67806	0.00276203	*Ebf2*	-1.31106	0.0380177
Cd34	-1.66261	0.000111783	*Kif21a*	-1.29354	0.00335546
Igfbp6	-1.63928	0.0324467	*Lamc1*	-1.28955	0.0100777
Aldh1a7	-1.56683	0.00597079	*Ogfrl1*	-1.2842	0.0489224
Kcnk1	-1.55923	0.00169676	*Ogfrl1*	-1.2842	0.0489224
Pmp2	-1.55216	0.00545035	*Amy1*	-1.28166	0.0122268
Olfr1137	-1.54521	0.00791402	*Lyzl6*	-1.28151	0.0251059
Foxb2	-1.52389	0.00377224	*Prdm5*	-1.26462	0.0405877
D230014K01Rik	-1.51836	0.000888305	*Tgif2lx*	-1.2614	0.00449781
Fez1	-1.49855	0.000119574	*Zfp354a*	-1.25921	0.0358394
Igf1	-1.49657	0.00160472	*2700097O09Rik*	-1.25583	0.00158081
Spink3	-1.49393	0.00811639	*Lims2*	-1.24776	0.0244591
Rhot1	-1.486	0.00222172	*9530058B02Rik*	-1.24652	0.00302437
Rarres2	-1.48103	0.0438235	*A930001N09Rik*	-1.23245	0.0351569
Abca8a	-1.47945	0.0213213	*4930547C10Rik*	-1.2251	0.00326749
Podn	-1.47056	0.0329607	*Rab33a*	-1.2221	0.0288482
1190002H23Rik	-1.46525	0.00738975	*Slc7a2*	-1.22177	0.0163603
Kcnd3	-1.45841	0.000983233	*Lifr*	-1.2123	0.00700647
Gucy1a3	-1.45573	0.00193329	*Apoc1*	-1.20872	0.00014886
Papolb	-1.4526	0.0114319	*Rbj*	-1.20447	0.0410994
Olfr1258	-1.44228	0.00459003	*4921517D22Rik*	1.20146	0.0237231
Gpx6	-1.44008	0.00395366	*She*	1.20723	0.00603411
Mlana	-1.42558	0.0127752	*Gjb6*	1.22419	0.0263954
Fhl1	-1.42104	7.67E-05	*Pld3*	1.2269	0.00102071
Zfp810	-1.42044	0.00305435	*Synpo2*	1.24448	0.00181895
Dpt	-1.40332	0.0230521	*LOC100044170*	1.25329	0.0312535
BC089491	-1.40325	0.0234	*Thrap2*	1.26598	0.00816893
Bglap-rs1	-1.39958	0.0259697	*Nts*	1.26854	0.0291884
Has2	-1.3985	0.000516123	*Snf8*	1.27172	0.00569249
Mertk	-1.38562	0.000466808	*4632417K18Rik*	1.27442	0.0481066
LOC100044204	-1.38151	0.0038226	*Prkcq*	1.28469	0.0372144
V1rg8	-1.37953	0.0293862	*Wasf2*	1.28897	0.0103153
Foxo1	-1.372	0.00955678	*Uroc1*	1.28898	0.0437403
Tmem159	-1.36099	0.000486166	*Mdk*	1.297	0.00558808
Tecta	-1.36057	0.0117076	*Slc5a1*	1.2994	0.0212787
Gucy1b3	-1.36033	0.0399826	*Olfr1350*	1.31063	0.00124012
Kcnk13	-1.36017	0.00859374	*Tmprss11f*	1.31098	0.0235681
Repin1	-1.35019	0.00347159	*Stat6*	1.31723	0.0109208
D11Bwg0517e	-1.34474	0.00226071	*Il3ra*	1.32523	0.0147557
Nope	-1.34461	0.00435024	*Tpm2*	1.32899	0.0175428

Gene	Fold change	P-value
Reep1	1.34405	0.0067372
Slc17a8	1.34712	0.0045408
Oas1e	1.3478	0.0054123
Kiss1	1.35257	0.0137431
AW554918	1.36053	0.0280153
Chodl	1.36935	0.0045297
Eif4e3	1.37453	0.0112004
Olfr1038	1.37554	0.0366871
Sh3kbp1	1.37565	0.0014579
EG432986	1.37725	0.0078885
V1rd2	1.37791	0.0050281
Htr1d	1.38189	0.0033634
Pfkl	1.38455	0.0047452
Casp1	1.39408	0.0153855
Casp14	1.39511	0.0010743
Pkm2	1.39859	0.0018724
Arhgdib	1.41896	0.0038286
Olfr1247	1.41963	0.0211009
Col5a2	1.41973	0.0060949
Slc2a1	1.42086	0.0040931
Fhl2	1.42415	0.0062281
Krt2-1	1.4256	0.0079384
Dr1	1.44053	0.000916
Car4	1.45069	0.0135643
Gsdmc1	1.45512	0.0296725
Tbc1d10c	1.46483	0.0009461
Asb2	1.4907	0.0296462
Egln1	1.50057	0.0006569
Fcer1a	1.52553	0.036528
Grap	1.53267	0.0043786
Cyp7b1	1.5374	0.0065935
Oas1d	1.54954	0.0010528
Slc38a5	1.58554	0.0127134
Lck	1.588	0.0250815
Mr1	1.65937	0.0025427
Serpinb3d	1.66572	0.0192279
BC117090	1.70551	0.0220388
Krt7	1.70771	0.001715
Cbln1	1.71743	0.0232234
Tgm5	1.72607	0.0002453
Serpinb3a	1.78082	0.0034267
Ms4a10	1.89451	0.0020406
Rbp2	1.96771	0.0001137
Krt6a	2.01919	0.0373743
Alox8	2.12963	0.0408879
H2-M2	2.13939	0.0203356
Fetub	2.66755	0.0005522
AA467197	2.70992	0.0307436
Ankk1	3.49322	0.0003154
Krt16	3.76846	0.011513

* Genes listed in order of fold change; most profoundly down-regulated gene at top
** Fold change in lymphedema model compared to non-operated control

Table S2. Changes in lymphedema tissue components due to linsitinib treatment

Tissue compnents *	Cross-sectional area of tissue component reduced by linsitinib (%)	Contribution of reduction to decreased cross-sectional tail area due to linsitinib (%)
Whole tail	27	100
Subcutaneous tissue	50	46
Dermal fibrosis	15	10
Epidermis	30	10
Keratin-rich region	30	5
Fat	40	4

* All analyses at day 17 post-surgery

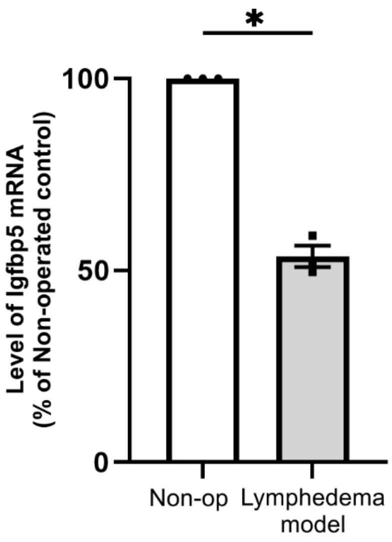

Figure S1. *Igfbp5* mRNA in the mouse lymphedema model. RT-qPCR analysis of *Igfbp5* transcript levels in mouse lymphedema model at day 84 post-surgery. The mean level of *Igfbp5* mRNA detected in non-operated control mice (Non-op) is defined as 100%. The levels of *Igfbp5* mRNA were normalized to the levels of *β-actin* mRNA. Graphs show mean ± SEM from three independent experiments, n=4 per/group in each experiment, * $p<0.0001$ (Student's t-test).

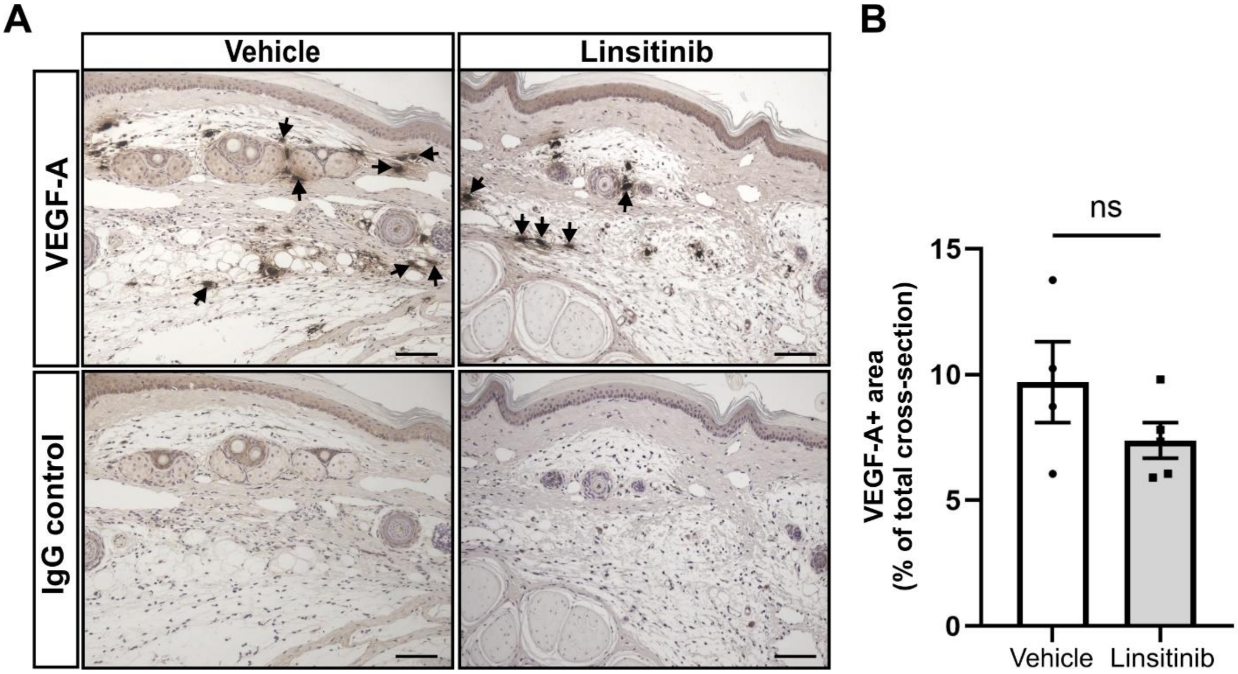

Figure S2. VEGF-A in the mouse lymphedema model. (A) Representative images of immunohistochemical staining for VEGF-A (top panels) in tail cross-sections from vehicle control and linsitinib-treated groups. Arrowheads indicate subsets of the VEGF-A signals (dark staining). Staining with IgG isotype concentration-matched control in serial sections is shown in the lower panels. Scale bars, 100 µm. (B) Percentage of VEGF-A+ area relative to total tail cross-sectional area. Graphs show mean ± SEM, n=4 for Vehicle and n=5 for Linsitinib, ns: not statistically significant (Student's t-test).

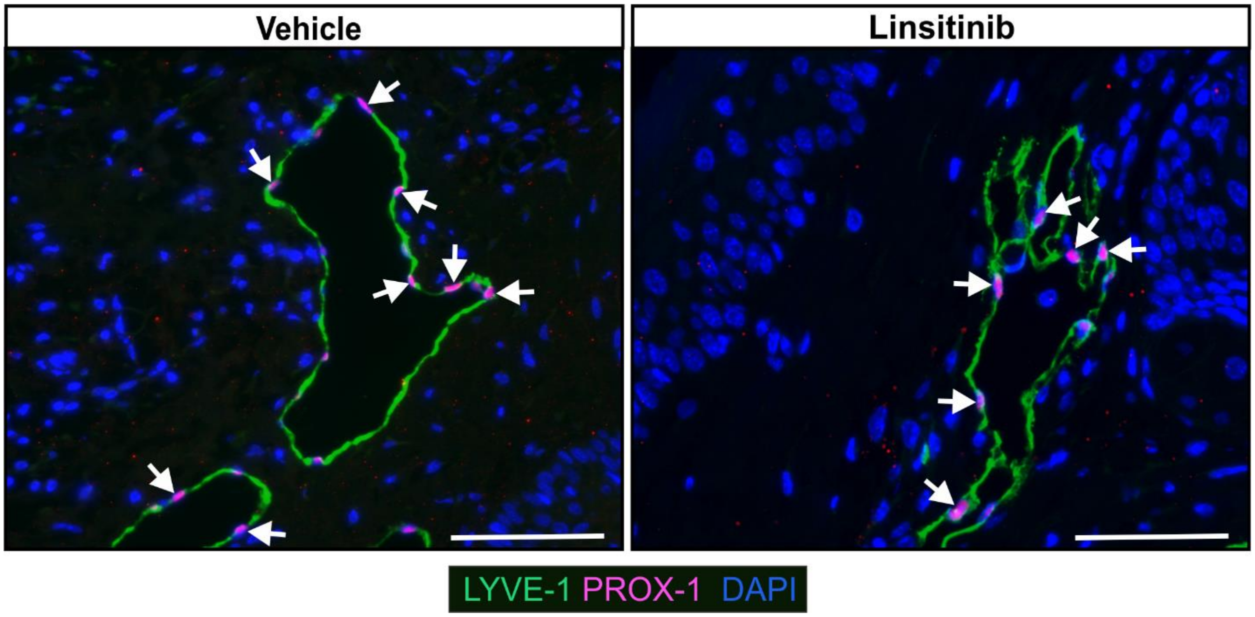

Figure S3. PROX-1 is localized on the nuclei of lymphatic endothelial cells in LYVE-1+ lymphatic vessels in the mouse lymphedema model. Representative images of immunofluorescence staining for PROX-1 (pink) and LYVE-1 (green) in tail cross-sections from linsitinib and vehicle control groups. Nuclei were stained with DAPI (blue). Arrowheads indicate PROX-1-positive nuclei. Scale bars, 50 μm.
